# Role of microbiota-gut-brain axis in natural aging-related alterations in behavior

**DOI:** 10.3389/fnins.2024.1362239

**Published:** 2024-04-18

**Authors:** Yingli Jing, Qiuying Wang, Fan Bai, Zihan Li, Yan Li, Weijin Liu, Yitong Yan, Shuangyue Zhang, Chen Gao, Yan Yu

**Affiliations:** ^1^China Rehabilitation Science Institute, China Rehabilitation Research Center, Beijing Key Laboratory of Neural Injury and Rehabilitation, and School of Rehabilitation Medicine, Capital Medical University, Beijing, China; ^2^Center of Neural Injury and Repair, Beijing Institute for Brain Disorders, Beijing, China

**Keywords:** aging, microbiota, short-chain fatty acids, behaviors deficits, inflammation, microbiota-gut-brain axis

## Abstract

**Introduction:**

Aging is a complex, time-dependent biological process that involves a decline of overall function. Over the past decade, the field of intestinal microbiota associated with aging has received considerable attention. However, there is limited information surrounding microbiota-gut-brain axis (MGBA) to further reveal the mechanism of aging.

**Methods:**

In this study, locomotory function and sensory function were evaluated through a series of behavioral tests.Metabolic profiling were determined by using indirect calorimetry.16s rRNA sequence and targeted metabolomics analyses were performed to investigate alterations in the gut microbiota and fecal short-chain fatty acids (SCFAs). The serum cytokines were detected by a multiplex cytokine assay.The expression of proinflammatory factors were detected by western blotting.

**Results:**

Decreased locomotor activity, decreased pain sensitivity, and reduced respiratory metabolic profiling were observed in aged mice. High-throughput sequencing revealed that the levels of genus Lactobacillus and Dubosiella were reduced, and the levels of genus Alistipes and Bacteroides were increased in aged mice. Certain bacterial genus were directly associated with the decline of physiological behaviors in aged mice. Furthermore, the amount of fecal SCFAs in aged mice was decreased, accompanied by an upregulation in the circulating pro-inflammatory cytokines and increased expression of inflammatory factors in the brain.

**Discussion:**

Aging-induced microbial dysbiosis was closely related with the overall decline in behavior, which may attribute to the changes in metabolic products, e.g., SCFAs, caused by an alteration in the gut microbiota, leading to inflammaging and contributing to neurological deficits. Investigating the MGBA might provide a novel viewpoint to exploring the pathogenesis of aging and expanding appropriate therapeutic targets.

## 1 Introduction

The world is currently facing a growing problem of population aging. The World Health Organization estimates that the proportion of the world’s population over 60 years old will shift from 12% in 2000 to 22% by 2050 (Fane and Weeraratna, 2020), population aging has become a major public health issue in China. By 2050, more than 400 million Chinese will reach the age of 65 or older, and 150 million will reach the age of 80 or older (Fang et al., 2015). Increasingly population aging has led to a shrinking proportion of the workforce and a significant increase in social spending on pensions, healthcare, and welfare protection, adding to the economic and social burden. Concomitantly, there is also an increased risk of many diseases associated with aging, such as cancer, cardiovascular disease, neurodegeneration, and many other common diseases, which greatly reduces the quality of life of the elderly (Campisi et al., 2019). Since aging is unavoidable, understanding the biological basis of aging and the associated mechanisms is important for healthy aging.

In recent years, the critical role of the gut microbiota in human aging has attracted more and more attention. A growing body of evidence suggests that the gut microbiota plays a critical role in human development, physiology, immunity, and nutrition (O'Hara and Shanahan, 2006). Throughout adult life, the microbial composition of the gut remains relatively stable, but changes dramatically with age. And microbial ecological dysbiosis occurs when the generated perturbations exceed the ability to restore symbiotic conditions of the microbiota and its host (Levy et al., 2017). This transition is characterized by a loss of diversity in the body’s flora. In the elderly, there is a decrease in the beneficial bacteria, such as *Mycobacterium anomalum* and *Bifidobacterium bifidum* (Santoro et al., 2018; Salazar et al., 2019), and an increase in the harmful bacteria, including *Aspergillus* and *Clostridium* (Claesson et al., 2011; Tavella et al., 2021). When they proliferate uncontrollably, the microorganisms and their metabolites may induce inflammatory responses, which may in turn contribute to the onset of chronic diseases in the elderly. Numerous possible mechanisms including inflammation, microbial dysbiosis and so on have been proposed to explain the origins of age-related neurodegenerative disorders (Sampson et al., 2016; Tansey et al., 2022; Grobler et al., 2023). These evidence support the view that inflammation and bacterial flora may be crucial treatment targets during the pathological and physiological process of senescence, which should be taken into account.

Also, changes in microbial composition are thought to play a key role in regulating the MGBA and influencing the brain and gut function (Borre et al., 2014; Sun et al., 2023). The MGBA is a bi-directional communication network between gut microbes and central neural system. Dysregulation of microbial ecology leads to dysfunction of MGBA signaling, which regulates host immune, metabolic, and neurological development and function. Studies have shown that transplantation of gut microbiota from aged rats into young rats resulted in a decreased neuronal functional activity in specific brain regions, and reduced synaptophysin expression in the young rats, all of which aligned with the characteristics observed in aged rats (Li et al., 2020). Another study found that the aged mice that received fecal transplants selectively reversed the immune response induce by aging, repaired aging-associated metabolic levels in the hippocampus, and improved learning and memory (Boehme et al., 2021). Cruz-Pereira et al. (2023) reported that age-dependent alterations of cecal metabolites affect deficits in social behavior. All of the above studies have demonstrated that ageing-induced microbial ecological dysfunctions might contribute to neurological deficits via metabolites.

In this study, we evaluated the locomotor activity, sensory function and cognitive level of young (3 month old) and aged (22 month old) female C57BL/6 J mice through a series of behavioral tests. The physiological functions, gut microbiota and their metabolites of young and aged mice were comparatively analyzed from the perspective of the MGBA. Our study focused on the alterations in the microbiota and metabolites induced by aging, and whether such alterations affect systemic inflammation and inflammation of related brain region through the MGBA to mediate abnormal behaviors.

## 2 Materials and methods

### 2.1 Animals

Ten young (3 month old) and eight aged (22 month old) female C57BL/6 J mice were obtained from the Center of Experimental Animals, Capital Medical University (Beijing, China). All mice were housed in an air-conditioned room where the temperature was 22 ± 2°C and relative humidity was 55% ± 10%, and followed a standard 12:12 light/dark cycle. Food and water were available *ad libitum*. The animal protocols were approved by The Animal Care and Use Committee of Capital Medical University (Approval No.: AEEI-2022-157).

### 2.2 Behavioral test

#### 2.2.1 Open field test

The open field test was carried out to evaluate locomotor activity. The apparatus was an opaque square chamber (50 × 50 × 40 cm) with a white floor. The center of the floor was illuminated at 100 lux. Each mouse was placed in the corner of the chamber. The total distance traveled (mm) and velocity (mm/s) were measured for 10 min using the Cleversys TopScanlite system.

#### 2.2.2 Rota rod test

Mice were placed on rotating drums (3 cm diameter) of an accelerating rotarod to evaluate motor coordination and balance. The speed of the rotarod accelerated from 0 to 40 rpm over a 5-min period. Latency to fall off the rotating rod was recorded with a 5-min cut-off time for three trials per day. The test was performed after 3-consecutive days of training.

#### 2.2.3 Gait analysis

Specific parameters of locomotion were quantified using the DigiGait Image Analysis System. For each test, at least 5 complete step cycles were recorded at a speed of 15 cm/s. A high-speed digital camera captured the movement of each paw and then the footage was analyzed using the DigiGait analysis software (DigiGait 12.4). The gait analysis evaluations were blinded to the experimenters.

#### 2.2.4 Hot plate test

The hot plate test was performed to evaluate sensitivity to a painful stimulus. Paw withdraw latency (PWL) was measured by placing the mouse’s hind paw on a radiant heat source. Mice were placed unrestrained in individual transparent plastic compartments (11 cm × 17 cm × 14 cm) and allowed to be stationary before the test. An infrared radiant heat source (25 W) was applied through a glass floor to the middle of the plantar surface of the hind paw until paw withdrawal was observed; the latency in the paw withdrawal response to the heat source was recorded as PWL. The intensity was set as 30% and cut-off limit of exposure was set as 30 s to avoid burning the skin of the mice.

#### 2.2.5 Von Frey hair test

Mice were placed in a transparent box with a wire mesh as the bottom surface and were given at least 30 min of adaptation time until they were calm before testing. Bilateral hind paw mechanical thresholds were determined by applying Von Frey filaments (Semmes–Weinstein monofilaments) with strengths ranging from 0.02 to 2.0 g (0.02, 0.04, 0.07, 0.16, 0.4, 0.6, 1.0, 1.4, 2.0 g). According to the “Up-Down” method (Dixon, 1980), 0.16 g filament was firstly applied to bilateral hind paws of mice until the filament was bent into S-shape, and the stimulation lasted for 3 s. If the mice showed rapid foot shrinkage or foot licking, it was recorded as a positive response and the intensity level was lowered by one level, and if it was a negative response, then the intensity level was raised by one level. Each measurement was taken about 5 min apart, and the final mechanical pain threshold was calculated.

### 2.3 Metabolic parameters

To measure the metabolic phenotyping, animals were transferred to metabolic phenotyping chambers (Mouse Promethion Continuous caging system; Sable Systems, Las Vegas, NV) individually. Gas sensors were calibrated before each run with 100% N_2_ as reference value zero. The incurrent flow rate was set at 2,000 mL/min and gases were sampled continuously for each cage, from multiple points within the cage (250 mL/min). Carbon dioxide (CO_2_) production and oxygen consumption were recorded for each mouse. The ratio of CO_2_ production over O_2_ consumption was calculated as respiratory exchange quotient (RQ). The Weir equation: Kcal/h = 60 × (0.003941 × VO_2_ + 0.001106 × VCO_2_) was used to calculate energy expenditure (EE). Values were calculated after application of algorithms using macros provided with the analysis software ExpeData.

### 2.4 Western blot analysis

Total protein was cleaved in lysis buffer (Beyotime, China) for 1 h. Tissue homogenates were then centrifuged at 14,000 g for 8 min at 4°C. The protein content of the supernatants were then analyzed by using a protein assay kit (BCA, Pierce, Rockford, IN, United States). Equal amounts of total protein (50 mg) were separated using 12% sodium dodecyl sulfate-polyacrylamide gel electrophoresis and transferred to a polyvinylidene difluoride membrane. Afterwards, the membranes were blocked with 5% skimmed milk in tris-buffered saline solution containing 0.05% Tween 20 (TBST) for 1 h and then incubated with antibody against Iba-1 (1:500, GeneTex, GTX100042), interleukin-1β (IL-1β; 1:1,000, Abcam, ab9722), tumor necrosis factor-α (TNF-α; 1:1,000, Abcam, ab6671), NeuN (1:1,000, Abcam, ab177487), NF-200 (1:1,000, Abcam, ab82259), Synapsin (1:1,000, Abcam, ab64581) at 4°C overnight. After 3 washes with TBST, the appropriate horseradish peroxidase-coupled secondary antibody was added and β-actin (1:1,000, Abcam, ab8227) was used as an internal control. The bands were visualized using enhanced chemiluminescence and images were acquired using the ChemiDoc MP system (Bio-Rad, Hercules, CA, United States). Relative band intensities were quantified using Image Lab4.1 (Bio-Rad, Hercules, CA, United States).

### 2.5 Multiplex cytokine assay

The blood samples from young and aged mice were analyzed by Cytometric bead array, as described previously (Zareie et al., 2017). The concentration of IL-23, IL-1α, IFN-γ, TNF-α, CCL2 (MCP-1), IL-12p70, IL-1β, IL-10, IL-6, IL-27, IL-17A, IFN-β, GM-CSF was measured by LEGEND plex™ flow-based 13-plex mouse inflammation panel kit from Biolegend (Fell, Germany; catalog number 740150). All the related reagents, the flow cytometry protocol, and the result analysis were implemented according to the manufacturer’s protocol. In brief, after a series of cytokine standard dilutions were prepared, beads coated with specific capture antibodies were mixed, respectively. Then 50 μL of the mixed captured beads, 50 μL of the plasma supernatant samples or standard dilutions, and 50 μL of phycoerythrin (PE) detection reagent were added sequentially to each tube. All the array tubes were gently mixed and incubated for 2 h at room temperature protected from light. After rinsing with wash buffer and centrifuging, 25 μL of detection antibody and 25 μL of SA-PE were added all the tubes, respectively. The samples were rinsed with 1 mL of wash buffer and centrifuged at 1,000 × g for 5 min. The bead pellets were resuspended in 300 μL buffer after discarding the supernatant. Analysis was performed employing BD accuri C6Plus cytometer (BD Biosciences, Heidelberg, Germany) and FlowJo software (FlowJo LLC, Ashland, OR).

### 2.6 Serum lipopolysaccharide binding protein analysis

The level of serum LBP was determined by using enzyme-linked immunosorbent assay (ELISA) kit (Cusabio Biotech Co., Ltd., Wuhan, China). As for ELISA, the procedure was in accordance with the instruction. Briefly, standards and plasma samples were added into the microplate onto which a monoclonal antibody specific for LBP was pre-coated. After washing away any unbound substances, an enzyme-linked polyclonal antibody specific for LBP was added into the microplate. Subsequently, all samples were reacted with the chromogenic solution and stop solution. The intensity of the color was measured on a plate reader (EnSpire; Perkin Elmer) at 450 nm.

### 2.7 16S rRNA gene amplicon sequencing

Fecal samples were collected in 2.0 mL sterile tubes, snap frozen in liquid nitrogen, and stored at −80°C for further analysis. The fecal samples were then placed in dry ice and sent to Shanghai Majorbio Bio-pharm Biotechnology Co., Ltd. (Shanghai, China) for 16S rRNA Gene Amplicon Sequencing and Bioinformatics and Statistical Analysis. Microbial genomic DNA was extracted using the OMEGA Stool DNA Kit (D4015-02, Omega Bio-Tek, Norcross, GA, United States) according to the manufacturer’s instructions. The quality of extracted DNA was examined by agarose gel electrophoresis, and quantified using QuantiFluor™-ST (Promega, United States). PCR amplification of the bacterial 16S rRNA genes V3–V4 region was performed using the forward primer 338F (5′-ACT CCT ACG GGA GGC AGC A-3′) and the reverse primer 806R (5′-GGA CTA CHV GGG TWT CTA AT-3′). After the individual quantification step, amplicons were pooled in equal amounts, and paired-end sequenced (2 × 300 bp) on an Illumina MiSeq platform according to the standard protocols. The raw reads were deposited into the NCBI Sequence Read Archive database (Accession Number: SRP485545).

Alpha and beta diversity analyses were performed using the diversity plugin in QIIME2. The Chao index and Ace index were employed to characterize alpha-diversity at the ASV level. Beta-diversity analysis was conducted through principle coordinate analysis (PCoA) using a weighted UniFrac dissimilarity matrix. Methods, including the Wilcoxon rank-sum test and LEfSe were employed to identify bacterial taxa with differing abundance between groups. Spearman correlation analysis was conducted to assess the correlation between important bacterial taxa and differential genes. Data analysis was carried out on the free online Majorbio Cloud Platform.^1^

### 2.8 Detection of fecal SCFA

Fecal samples were weighed and 20 mg of the samples were transferred into a 2 mL EP tube. Then 1 mL phosphoric acid (0.5% v/v) solution was added into the EP tube, followed by vortexing for 10 min, and ultrasonic wave treatment for 5 min. Then 0.1 mL supernatant was transferred into a 1.5 mL centrifugal tube, followed by addition of 0.5 mL MTBE (containing internal standard) solution, vortexing for 3 min, and ultrasound treatment for 5 min. After that, the samples were centrifuged for 10 min at 12,000 r/min at 4°C. Then the supernatant was analyzed by using a gas chromatography–mass spectrometry (GC–MS/MS 7890B7000D; Agilent Technologies Inc.) on a silica capillary column (DB-FFAP, 30 m × 0.25 mm × 0.25 μm, Agilent J&W) under the following conditions: injected sample size, 2 μL, splitless; injector temperature at 200°C; initial oven temperature at 95°C, held for 1 min, raised to 100°C at a rate of 25°C/min, raised to 130°C at a rate of 17°C/min, held for 0.4 min, raised to 200°C at a rate of 25°C/min, and held for 0.5 min. Helium was used as a carrier gas at 1.2 mL/min. The main mass spectrometry conditions were as follows: ion source, EI; transfer line temperature, 230°C; ion source temperature, 230°C; quad temperature, 150°C, electron energy, 70 eV; scan mode, MRM; and solvent delay, 2.4 min. The quantification of fecal metabolites was carried out by using Metware Biotechnology Co., Ltd. (Wuhan, China).

### 2.9 Statistical analyses

Data were presented as means and standard error of the mean and examined using SPSS 17.0 (SPSS Inc., Chicago, IL, United States). The statistical significance was determined using one-way ANOVA followed by Tukey’s multiple comparisons test and two-tailed unpaired t tests were used for comparisons between two groups. Spearman correlation was used to analyze the correlation. We performed all statistical tests using GraphPad Prism 9.0 Software (San Diego, CA). A significance level of *p* < 0.05 was considered statistically significant.

## 3 Results

### 3.1 Aged mice exhibited decreased locomotor activity and sensory function

**Figure 1:**
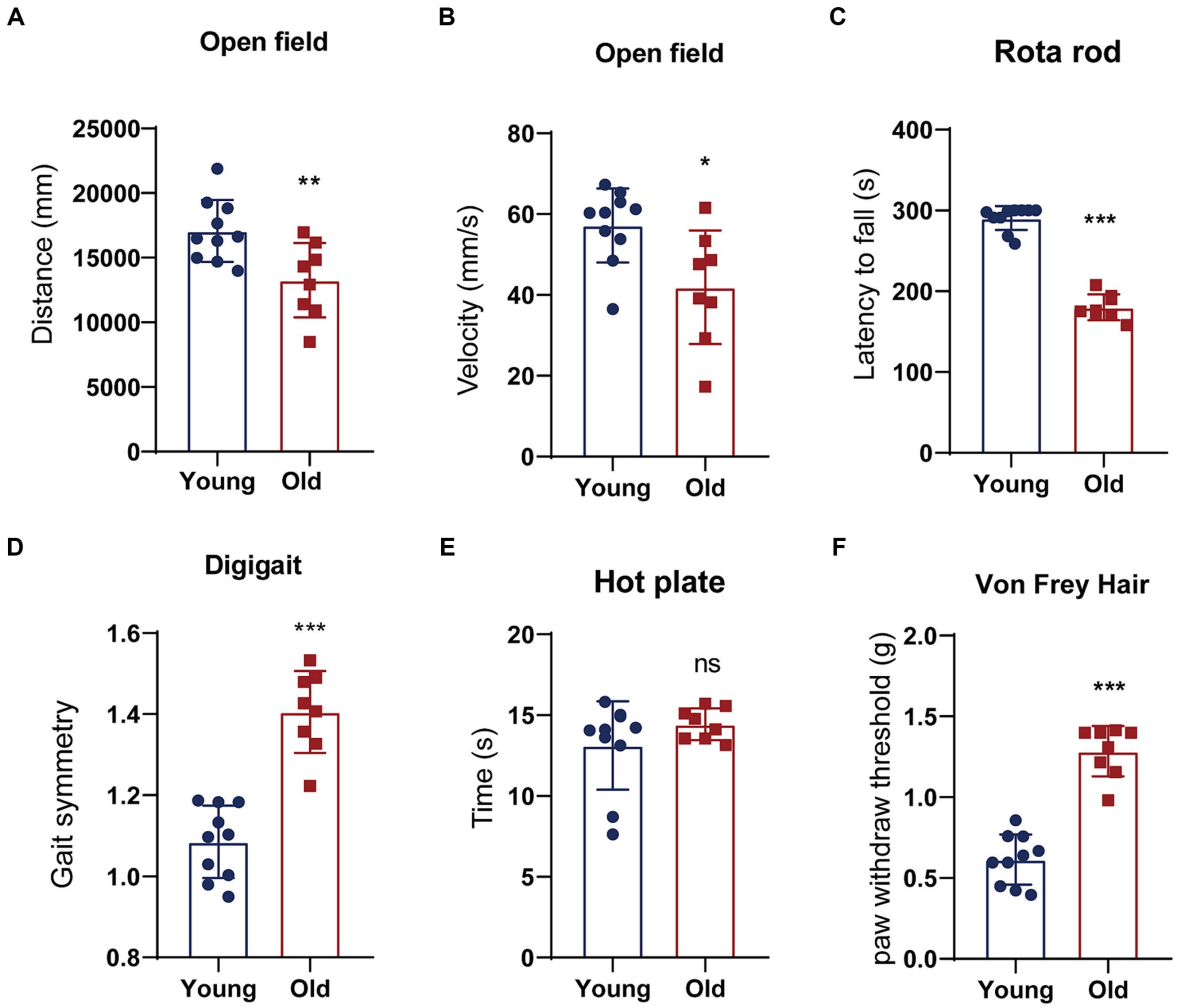
Behavioral tests of young and aged mice. **(A,B)** Distance traveled or the average velocity during the open-field test; **(C)** latency to the time point when mice fell off a rotating rod (s) in the rotarod test; **(D)** gait analyzed using an automated treadmill (DigiGait). **(E)** A latency to withdrawal from a focused heat stimulus. **(F)** The mechanical sensitivity was assessed with different forces of Von Frey filaments. ^*^p < 0.05; ^**^p < 0.01; ^***^p < 0.001.

Aging is a natural and time-dependent physiological process that can lead to a decrease in overall function. Therefore, we performed open field, rotarod, DigiGait test to assess locomotory in aged and young mice. An open field test was used for measuring spontaneous locomotor activity. Young mice exhibited greater total distance traveled and faster average velocity during the open field test (Figures 1A,B). Meanwhile, we conducted rotarod test to evaluate motor coordination and balance between two groups. The result showed that young mice had remarkably more time spent on the rotarod in comparison to the aged mice (Figure 1C). DigiGait is a sensitive test to trace subtle changes in the gait patterns in mice. The data revealed that with aging, the symmetry of mice gait deteriorates (Figure 1D). We also investigated sensory function using thermal and mechanical sensitivity testing. Hind paw response was examined to a radiant heat source to test for thermal sensation. Aged mice performed similarly to young mice in latency to withdrawal in hot plate test (Figure 1E). The mechanical sensitivity was assessed with different forces of Von Frey filaments. Mice in young and aged groups show significant different mechanical allodynia in response to filaments (Figure 1F). Cognitive behavior is also evaluated using novel object recognition test and spontaneous alternation in Y-maze. Although corresponding parameters between young and old mice showed certain differences, the differences were not statistical significant (data not shown).

### 3.2 Aged mice exhibited decreased metabolic profiling

**Figure 2:**
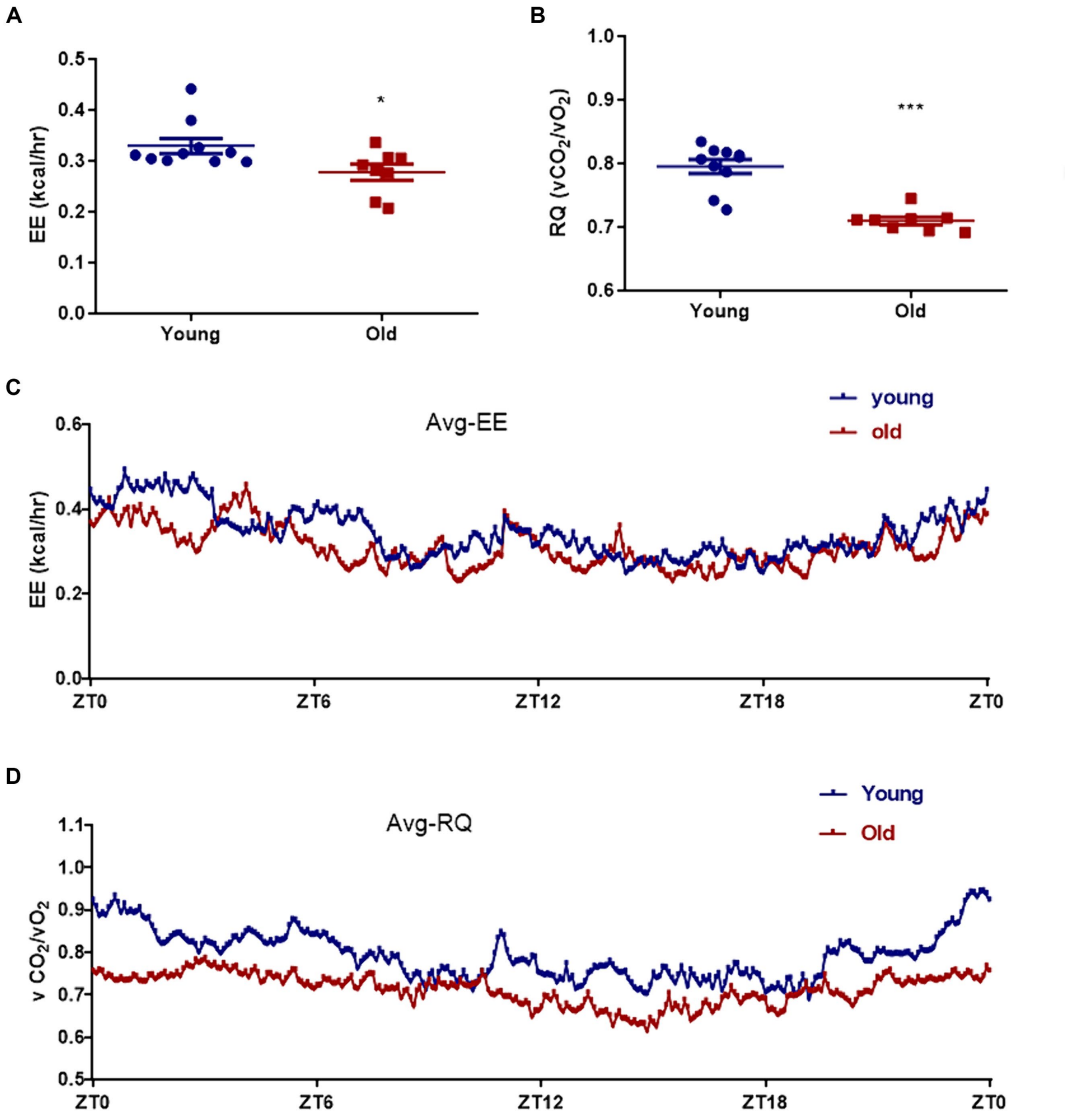
Effect of aging on metabolism profiling. Energy expenditure (EE) **(A)** and respiratory quotient (RQ) **(B)** were measured in both young and age mice. The mean energy expenditure (Avg_EE) **(C)** and the mean respiratory quotient (Avg_RQ) **(D)** were measured every 5 min for 24 h. ^*^p < 0.05; ^***^p < 0.001.

Aging is associated with metabolic alteration. The metabolic parameters were assessed over a 24 h period between young and old mice. Circadian energy expenditure and food consumption were determined by using metabolic cages. Indirect calorimetry revealed that the average energy expenditure was significantly elevated in young mice when compared with aged mice (Figures 2A,C). As shown in Figures 2B,D, the average respiratory exchange quotient (RQ) values were higher in young mice than that in aged mice. However, the difference in direct respiratory metabolism between young and old mice was significantly greater than the difference in energy expenditure. Energy expenditure is directly related to body weight, and elderly mice weigh more than young mice. Respiratory metabolism is directly related to motor function, and compared to aged mice, young mice have greater motor abilities.

### 3.3 Aged mice altered gut microbiota profiles

**Figure 3:**
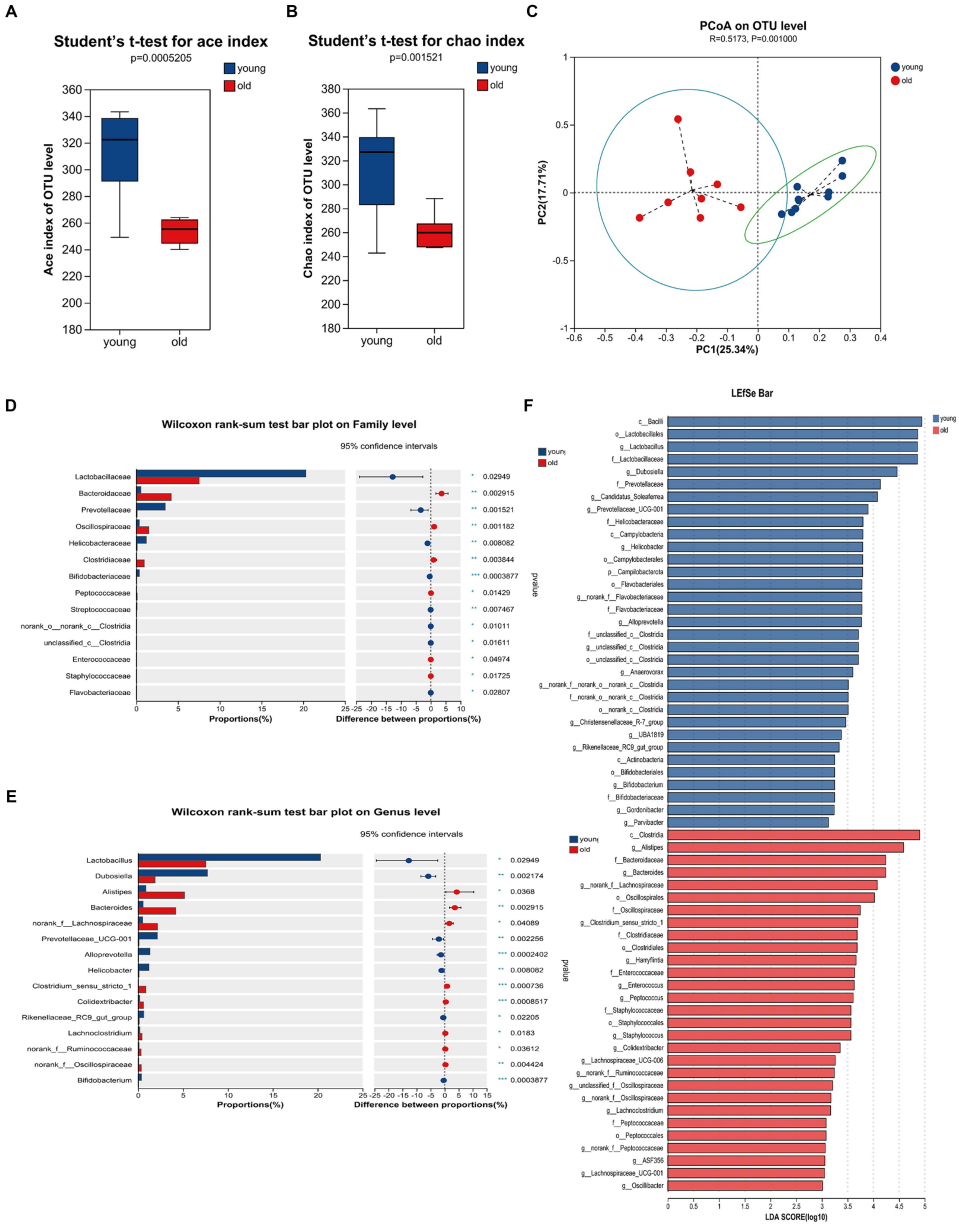
Gut microbiome composition profiles differ between young and aged groups. Comparison of ace index **(A)** and chao index **(B)** based on ASV levels in the two groups. **(C)** Scatter plots of principal coordinate analysis (PCoA) scores showing the similarity of the bacterial communities based on the Bray-Curtis distance. **(D)** Quantitative analyses of the relative abundances of bacterial at the family level. **(E)** Quantitative analyses of the relative abundances of bacterial at genus level. **(F)** LEfSe analysis showed significant differences in bacterial abundances in the fecal microbiota between young and aged groups. Linear discriminant analysis scores (log10) > 3 and p < 0.05 were listed. ^*^p < 0.05; ^**^p < 0.01; ^***^p < 0.001.

Advancing age is accompanied by alterations in the intestinal microbiome, characterized by the loss of protective commensal microorganisms induced by internal and external factors such as the accumulation of DNA damage, medications, chronic health conditions and so on (Conway, 2021; Ghosh et al., 2022). To examine the effects of age on gut microbiota composition, we performed 16S rRNA (V3 + V4 regions) gene sequencing on microbiota in mice feces. As shown in Figures 3A,B, there were significant differences in the ace and chao indices between young and aged mice, suggesting that aging had a marked impact on the richness of microbiota. To measure the degree of similarity between microbial communities, β-diversity was further evaluated by Bray-Curtis principal coordinate analysis (PCoA; Figure 3C). Young group clustered distinctly from the aged group, moreover, ANOSIM of β-diversity analysis indicated robust differences in the structure of the gut microbiota between the two groups. Compared to the aged group, young mice showed distinct microbiota up to the family level. There was an increase in the abundance of *Lactobacillaceae*, *Prevotellaceae*, *Helicobacteraceae* and *Bifidobacteriaceae* in young mice; whereas, significant alterations in the microbiota were accompanied by lesser but consistent alterations in minor microbiota, including *Streptococcaceae* and *Flavobacteriaceae*. The abundance of *Bacteroidaceae*, *Oscillospiraceae*, and *Clostridiaceae* were increased in aged mice, accompanied by lesser but consistent alterations in minor microbiota, including *Peptococcaceae*, *Enterococcaceae* and *Staphylococcaceae* (Figure 3D). As shown in Figure 3E, the genus level analysis demonstrated that the relative abundance of *Lactobacillus*, *Dubosiella*, *Bifidobacteriales*, *Prevotellaceae*_UCG-001, *Alloprevotella*, *Helicobacter*, and *Rikenellaceae*_RC9_gut_group, *Bifidobacterium* was significantly decreased, whereas the relative abundance of *Alistipes*, *Bacteroides*, *Clostridium*_sensu_stricto_1, *Colidextribacter*, *Lachnoclostridium* was distinctly increased in aged mice. The species and communities making significant contributions to the differences between the two groups were analyzed by linear discriminant analysis (LDA) effect size (LEfSe). We adopted the threshold logarithmic LDA score at 3.0 and *p* < 0.05 to determine the key taxonomic differences between both groups. Figure 3F shows significant differences in genera. In addition, *Bacteroidaceae* and *Bacteroides* were the LPS-producing bacteria with high impact (LDA score > 4) in the aged mice, Figure 3F showed the differences of the LPS-producing bacteria between young and aged groups, and the abundance of *Bacteroidaceae* and *Bacteroides* in the aged mice was evidently predominant. Also, serum LPB level was significantly increased in aged mice when compared to young mice (Supplementary Figure S1). In general, these overall alterations of microbiota composition with aging exacerbated pathological and physiological changes in the elderly.

### 3.4 Correlation between microbiota and overall activity

**Figure 4:**
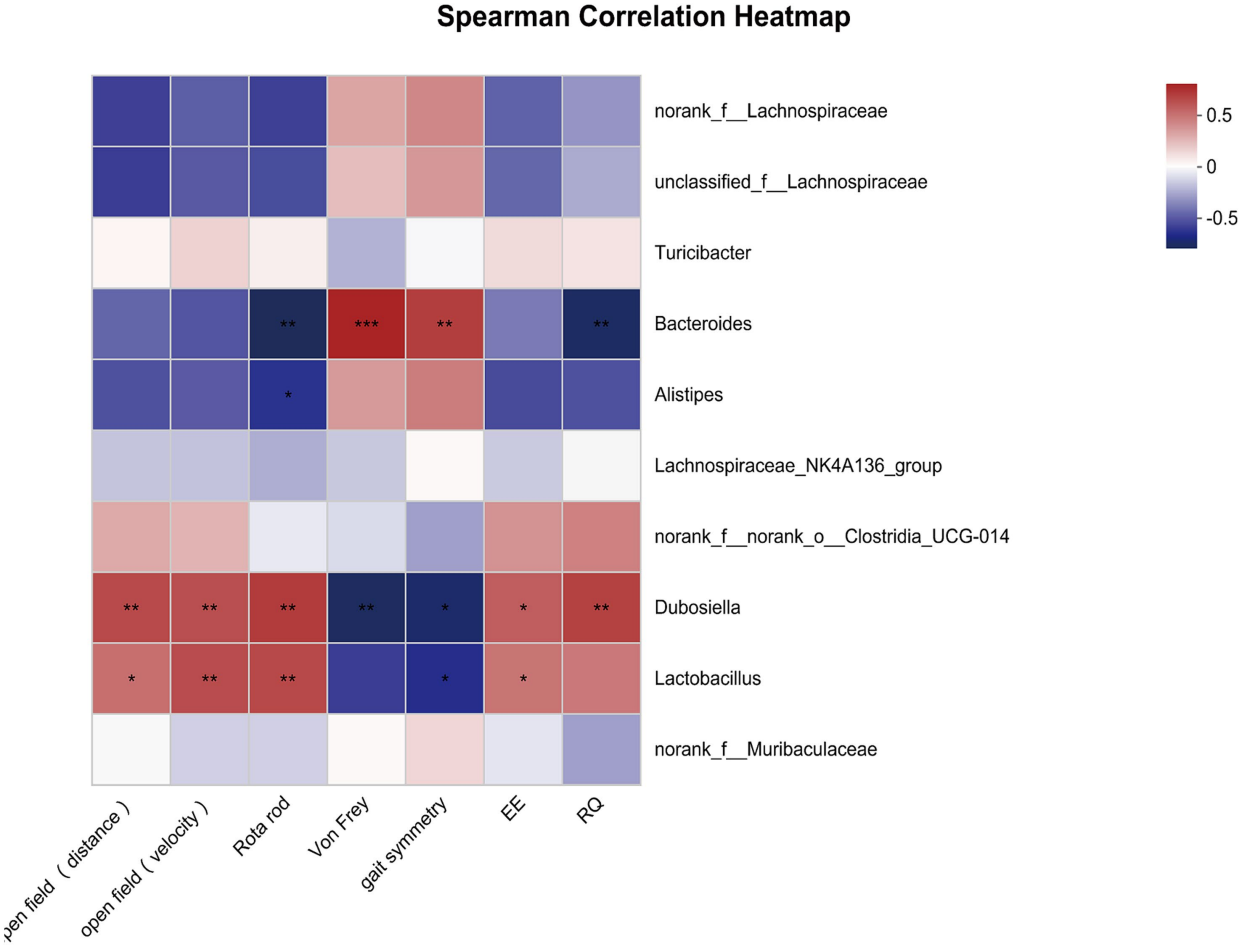
Correlations between overall activity and community composition. Correlation heatmap analysis of overall activity (the total distance and mean velocity in open field, time spent on the rotarod, paw withdraw threshold, gait symmetry scores, circadian energy expenditure and respiratory exchange quotient) on the community composition of the young and old mice at the genus level. ^*^p < 0.05; ^**^p < 0.01; ^***^p < 0.001.

Next, we estimated whether certain bacterial genus was correlated with overall activity. As shown in Figure 4, the total distance in open field was positively correlated with *Dubosiella* and negatively correlated with *Alistipes*. The mean velocity in open field was positively correlated with *Dubosiella* and *Lactobacillus*. Similarly, time spent on the rotarod was positively correlated with *Dubosiella* and *Lactobacillus*, and then was negatively correlated with *Bacteroides* and *Alistipes*. Inverse relationships were observed between microbiota and pain threshold / coordination. *Bacteroides* was positively correlated with paw withdraw threshold and gait symmetry scores; *Dubosiella* were negatively correlated with paw withdraw threshold and gait symmetry scores. Gait symmetry scores were still positively correlated with *Lactobacillus*. In addition, circadian energy expenditure showed a positive relationship with *Dubosiella* and a negative relationship with *Alistipes*, *Bacteroides*, *Turicibacter* and *unclassified_f__Lachnospiraceae*. Similarly, respiratory exchange quotient showed a positive relationship with *Dubosiella* and *Lactobacillus*, and a negative relationship with *Alistipes* and *Bacteroides*. It seems that the certain bacterial genus could provide a degree of predictive power as to senescence, which might represent potential targets for intervention in order to delay aging or promote healthy aging.

### 3.5 Aged mice altered fecal SCFA contents

**Figure 5:**
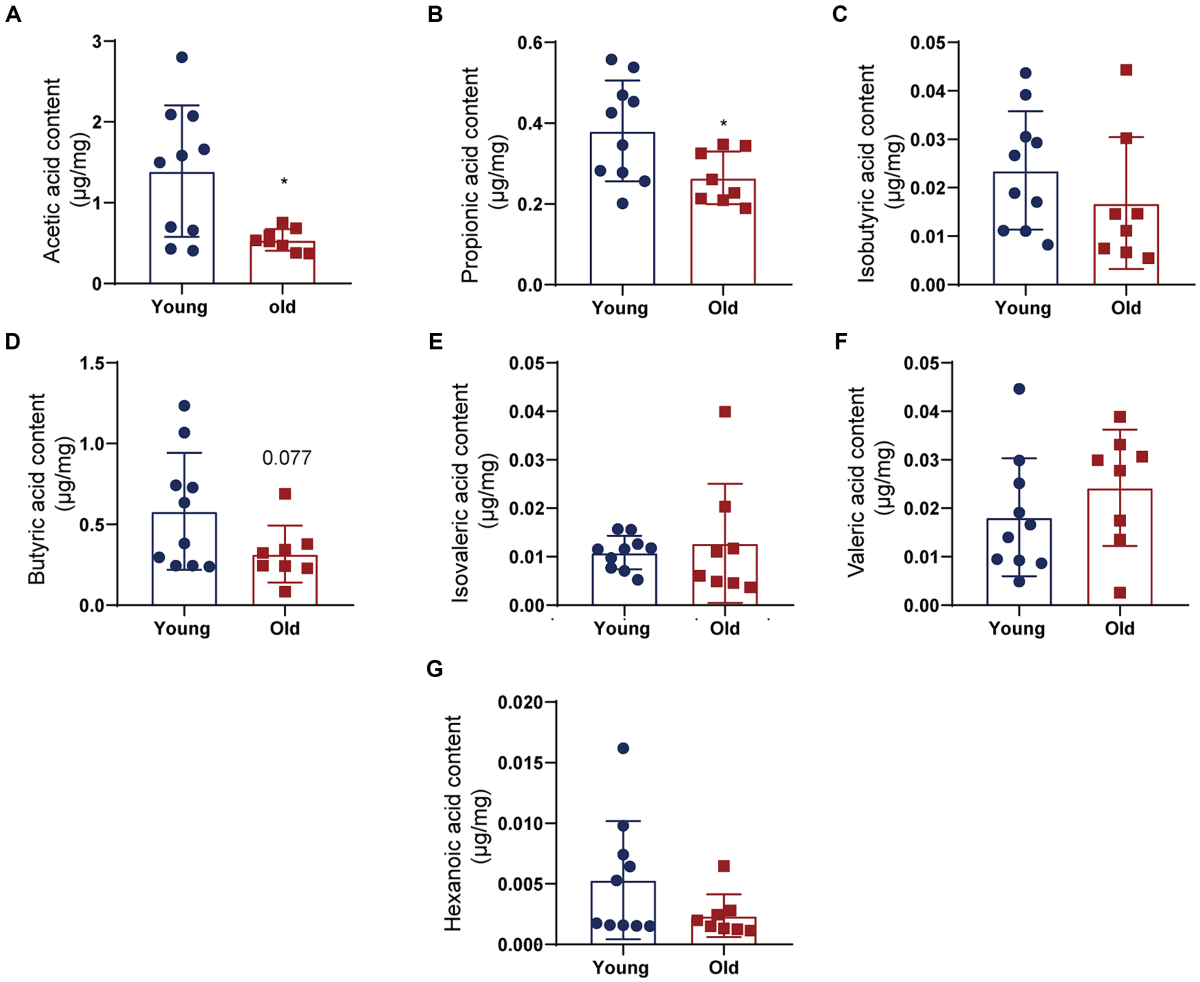
Fecal SCFA contents differ between young and aged groups. Quantitative analyses of SCFA levels including acetic acid (AA) **(A)**, propionic acid (PA) **(B)**, isobutyric acid (IBA) **(C)**, butyric acid (BA) **(D)**, isovaleric acid (IVA) **(E)**, valeric acid (VA) **(F)**, and hexanoic acid (HA) **(G)** were performed by using a gas chromatography–mass spectrometry between control and SCI groups. ^*^p < 0.05.

Studies have shown that certain probiotics colonized in the intestine, such as *Dubosiella* and *Lactobacillus,* are involved in regulating gut microbiota metabolism and production of SCFAs (Lv et al., 2020; Ye et al., 2021; Tian et al., 2022). However, it is unclear whether SCFA levels were altered in aged mice. GC–MS was used to measure SCFA contents, such as acetic acid (AA), butyric acid (BA), hexanoic acid (HA), propionic acid (PA), valeric acid (VA), isobutyric acid (IBA), and isovaleric acid (IVA; Figure 5). Compared to young mice, aging induced downregulation of AA, PA, and BA, decreasing by 61.6, 30.5 and 45.6%, respectively (Figures 5A,B,D). Among SCFAs, AA level in the examined fecal SCFAs changed most significantly. There were no significantly different in the level of IBA, IVA, VA and HA contents between young and old groups (Figures 5C,E–G). The data demonstrated that aging-induced microbiota dysbiosis might mediated by alteration of fecal SCFAs.

### 3.6 Aged mice showed elevated expression of pro-inflammatory cytokine in blood sample

**Figure 6:**
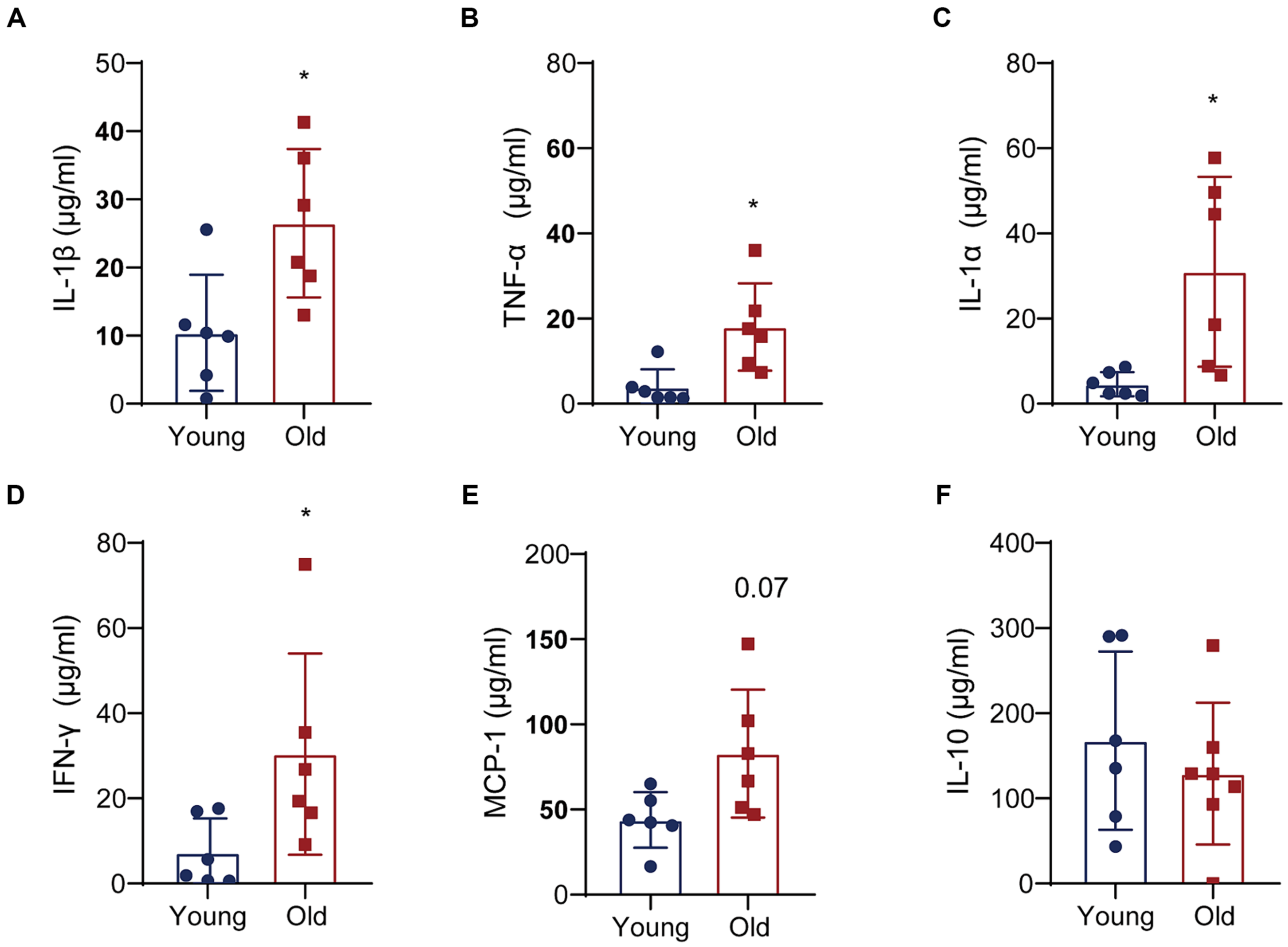
Aging promotes the expression of pro-inflammatory cytokines. Quantitative analyses of 13 cytokines in the colons were performed by multiplex enzyme immunosorbent assay. Quantitative analyses of IL-1β **(A)**, TNF-α **(B)**, IL-1α **(C)**, IFN-γ **(D)**, MCP-1 **(E)** and IL-10 **(F)** levels between two groups. ^*^p < 0.05.

To further elucidate the molecular interactions between aging-induced microbial dysbiosis and inflammaging, we detected cytokines in the circulation to evaluate peripheral inflammation. Levels of 13 cytokines were analyzed using cytometric bead array in serum. A number of pro-inflammatory cytokines in aged mice was upregulated, including IL-1β, TNF-α, IL-1α and IFN-γ, when compared with that in young mice (Figures 6A–D). There was an increasing trend in the expression of MCP-1 (Figure 6E) and a reducing trend in the expression of IL-10 (Figure 6F) in aged mice vs. young mice, which showed no statistical difference between two groups. In addition, no significant changes were found in IL-23, IL-12p70, IL-6, IL-27, IL-17A, IFN-β, GM-CSF following aging (data not shown).

### 3.7 Inflammation increased in the brain of aged mice

**Figure 7:**
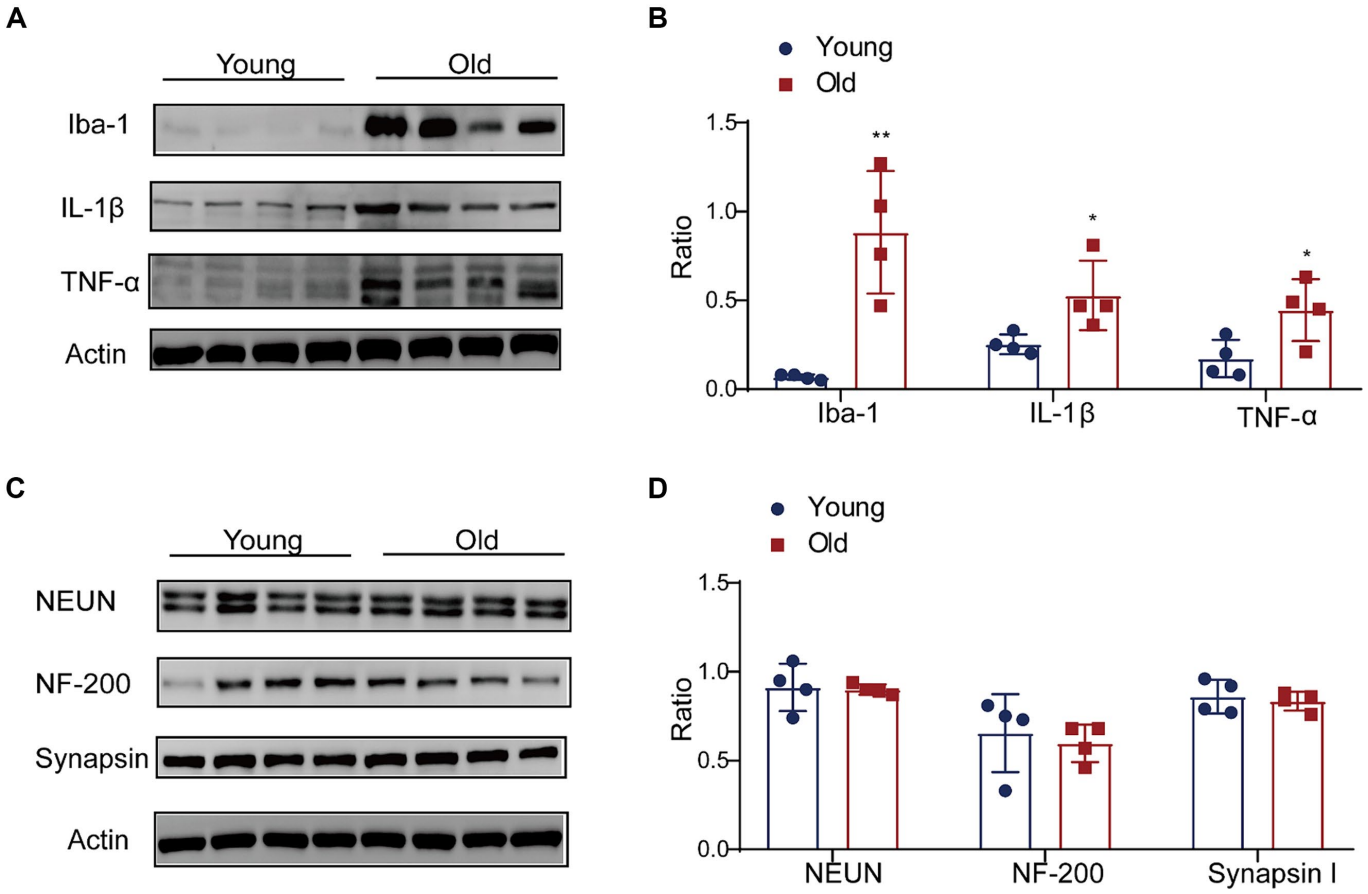
Effect of aging on expression of proinflammation cytokines in brain. **(A)** The expressions of Iba-1, IL-1β and TNF-α were detected by western blot; **(B)** the relative amounts of Iba-1, IL-1β and TNF-α were semi-quantified; **(C)** the expressions of NEUN, NF-200 and Synapsin were detected by western blot; **(D)** the relative amounts of NEUN, NF-200 and Synapsin were semi-quantified. ^**^p < 0.01; ^*^p < 0.05.

The gut microbiota is a crucial mediator for communicating between the gut and brain by regulating the function and maturation of microglia (Matcovitch-Natan et al., 2016; Thion et al., 2018; Mossad et al., 2022). We detected the alterations of microglia and pro-inflammatory cytokines in the brain with aging. A higher Iba-1 level was observed in the brain cortex (Figures 7A,B). Additionally, the expressions of IL-1β and TNF-α in the aged brain cortex were significantly increased when compared with young group (Figures 7A,B). In the aged brains, the produce of pro-inflammation cytokines negatively affected neurogenesis and function of synapase in mice (Sierra et al., 2010; Wu et al., 2013). We also detected the level of NF, Synapsin and NeuN. The results revealed that the expression of neural related proteins in the aged brain showed a decreasing trend, but there was no significant difference between groups (Figures 7C,D).

## 4 Discussion

The pathological and physiological changes of aging may be the result of the interaction of multiple factors, which have been implicated with various age-related disorders. A growing number of researchers were focusing on aged-related changes of MGBA concomitant with microbial metabolites, inflammation and behaviors. In the current study, we demonstrate that aging is related with significant change in the composition and abundance of the gut microbiota coupled with physiological alterations and behavioral abnormalities.

Alterations in gut microbiota composition gradually occur with advancing age, which characterized by a state of microbial dysbiosis that could result in or exacerbate disease (Biagi et al., 2016; Du et al., 2021). The hallmark of this shift was the loss of bacterial diversity. In our study, the abundance of specific genera, such as *Lactobacillus*, *Dubosiella*, and *Bifidobacteriales*, was markedly decreased in aged mice, suggesting a loss of relative abundance of beneficial commensal microbes with senescence. Significant changes were accompanied by the expansion of *Alistipes*, *Bacteroides* and other opportunistic microbiome such as *Clostridium_sensu_stricto*_1, *Colidextribacter* and *Lachnoclostridium*. In addition, aging induced the upregulation of LPS-producing bacterial such as *Bacteroidaceae* and *Bacteroides*, which are at lower abundance in young mice. It has been demonstrated that a high abundance of *Alistipes*, *Bacteroides*, *Clostridium_sensu_stricto*_1, *Colidextribacter* and *Lachnoclostridium* was associated with dysbiosis, oxidative stress, obesity, chronic inflammation and neurodegenerative diseases, indicating that these microbes may increase the risk of the onset of chronic diseases and inflammation (Zafar and Saier, 2021; Cai et al., 2022; Pang et al., 2022; Liu et al., 2023; Yang et al., 2023).

Perturbations in gut homeostasis and microbiome are considered as contributors of increased infection and inflammations. In elderly individuals and in laboratory aged animals, circulating pro-inflammatory factors including immune response and inflammation have been reported (Man et al., 2014; Mabbott, 2015; Scott et al., 2017). Similarly, in our study, the serum levels of pro-inflammatory cytokines (IL-1α, IL-1β, TNF-α, and IFN-γ) and LBP were upregulated in aged mice, which was the main feature of physiological aging. Scott et al. (2017) reported that the plasma levels of IL-6, IL-1β, and TNF-α were increased in aged mice, which was correlated positively with gut permeability. IL-1α and IFN-γ also plays an important part in inflammation and age-related disease (Acosta et al., 2013; Tan et al., 2021). Serum concentrations of LBP were approximately two times higher in aged mice than in young mice which responded bacterial LPS leakage into the bloodstream to accelerate inflammaging (Kim et al., 2016; Parker et al., 2022). Indeed, circulating low-grade inflammation (inflammaging) caused the uncontrolled development of chronic and degenerative diseases including Alzheimer’s disease (AD) and Parkinson’s disease (Bleve et al., 2023). More and more evidence from human samples and animal models supported the involvement of inflammation in onset or progression of Parkinson’s disease (Pajares et al., 2020). It was found that AD patients present with increased levels of pro-inflammatory cytokines, which was closely associated with AD risk genes (Leng and Edison, 2021). The levels of systemic inflammation and inflammatory signaling pathways in the brain were both elevated in age-related neurodegenerative disorders (Pajares et al., 2020; Leng and Edison, 2021). Circulating pro-inflammatory cytokines might be directly or indirectly involved in the damage of central nervous system (CNS) by amplifying the immune response and further result in the inflammation of CNS. Then, the causal relationship and exact triggering mechanism between neurodegenerative disorders and inflammation remained unclear.

In the present study, apart from systemic inflammation, we also detected the expression of inflammatory factors in the cerebral cortex. A higher expression of Iba1 (marker of microglia) and cytokines (TNF-α and IL-1β) was detected in the aged cerebral cortex. In the brain, microglia serve as the sentinels, which are regarded as key players in CNS homeostasis (Socodato et al., 2020; Castellani et al., 2023). High levels of reactive oxygen species and pro-inflammatory cytokines produced by microglia may be critical factors in neurodegeneration in aging human and mice brains (Marschallinger et al., 2020). Recently, the MGBA has received increasing attention in aging research. Aging causes disruption of gut microbiota and a decline in brain function. Recent studies indicated that age-associated microbiota dysbiosis caused microbe-associated molecular patterns such as microbiota and microbial metabolites entering the circulation which contribute to inflammaging (Thevaranjan et al., 2018). In addition, age-related microbiota alterations were not present in aged TNFα-deficient mice (Thevaranjan et al., 2018). Taken together, the data suggested that a potential feedback loop exists between inflammaging and microbial dysbiosis.

Aging is a complex process primarily propelled by the aggregation of DNA damage, resulting in a series of epigenomic, immunological, metabolic, and physiological changes which lead to the progressive functional deterioration and the onset of chronic diseases (Tian et al., 2022). In our study, reductions in respiratory metabolic spectrum and overall locomotor activity were observed in aged mice. Furthermore, motor function, sensory function, and coordination function were mainly positively correlated with *Dubosiella* and *Lactobacillus*, and then were negatively correlated with *Bacteroides* and *Alistipes*. Recent investigate showed that microbiota modulates behavioral and physiological abnormalities associated with neurodevelopmental disorders (Hsiao et al., 2013). Zeng et al. (2023) found that gut microbiota of old mice worsens neurological outcome after brain ischemia via increased valeric acid and IL-17 in the blood. Medina-Rodriguez et al. (2023) found that the microbial effect was dependent on the presence of Th17 cells in the recipient, which plays a key role in regulating behavioral changes induced by the microbiome of depressed patients. Zhu et al. (2023) reported that specific Lactobacillus species affected stress-induced social-avoidance behavior by involving in T cell differentiation mediated immune response. Combined with our research and published literatures, microbiota affects behavioral changes mainly or partly involved in immune response.

*Dubosiella* and *Lactobacillus* are superior genus of bacteria with significant differences between young and old mice. Also, *Dubosiella* and *Lactobacillus* are intestinal SCFA-producing bacteria (Lv et al., 2020; Ye et al., 2021; Tian et al., 2022). In aged mice, we also observed that main SCFAs (e.g., AA, PA) was significantly downregulation. Our previous study reported that SCFAs improved neurological recovery by inhibiting microglia activation and NF-κB signaling pathway in spinal cord injured mice (Jing et al., 2023). Sadler et al. (2020) found the substantial impact of SCFAs on microglial activation and immune cell infiltration in experimental stroke model. Long-term SCFA supplementation exerted neuroprotective effect in terms of restraining inflammatory response and hippocampal neuronal apoptosis in a model of chronic cerebral hypoperfusion (Xiao et al., 2022). Accumulating studies showed that SCFAs have profound influence on CNS by mediated immune function and suppressing inflammation (Silva et al., 2020; Dong and Cui, 2022). In the present study, apart from the decrease of SCFAs in aged mice, we also detected the increase of inflammatory factors in peripheral blood and cerebral cortex. It is possible that with aging changes in the composition of microbiota cause a decrease in key bioactive microbial metabolites such as SCFAs, leading to an increase in inflammation levels in the host. Subsequently, further experiments such as antibiotic intervention, fecal microbiota transplantation intervention, and SCFA supplementation will be conducted to determine whether microbiota was involved in the aging process by SCFAs mediating neuroinflammation. Further research will use probiotics or drugs targeting microbiota or metabolites for delaying the senescent process.

The microbiome and CNS have bidirectional communication via a variety of routes including enteric nervous system, vagus nerve system, endocrine system and metabolites (Kigerl et al., 2020). With aging, the composition and abundance of gut microbiota changed, leading to dysregulated microbial ecology and altered metabolites. These alterations mediated by microbiota further regulated immune activation, increased systemic inflammation and inflammation of related brain region via MGBA, which in turn destabilized the ecology of the gut microbiota and further aggravated the aging-associated behavioral decline and metabolic attenuation, and thereby increasing the risk of the onset of chronic diseases and inflammation.

In this study, the differential bacteria detected between young and aged mice are not fully aligned with previous literature (Man et al., 2014; Shin et al., 2021). The discrepancy might be related with age, gender, diet, and even breeding environment of animals (Shin et al., 2021; Russell et al., 2022; Wang et al., 2022). It is well established that in adulthood, microbiota of male and female mice varies (Wang et al., 2022). However, there is almost no literature studying on the differences in gut microbiota among aged mice based on sex. In this study, only female young and aged mice were used. Future studies will incorporate both sex, which is beneficial for parallel comparison of mouse microbiota in the same age and sex.

## Data availability statement

The data presented in the study are deposited in the NCBI Sequence Read Archive database, accession number SRP485545.

## Ethics statement

The animal study was approved by the Animal Care and Use Committee of Capital Medical University (Approval No.: AEEI-2022-157). The study was conducted in accordance with the local legislation and institutional requirements.

## Author contributions

YJ: Conceptualization, Funding acquisition, Writing – original draft, Data curation, Investigation, Project administration, Writing – review & editing. QW: Data curation, Investigation, Methodology, Writing – original draft, Writing – review & editing. FB: Investigation, Writing – review & editing. ZL: Software, Writing – review & editing. YL: Formal analysis, Investigation, Writing – review & editing. WL: Formal analysis, Investigation, Writing – review & editing. YYa: Data curation, Investigation, Writing – review & editing. SZ: Investigation, Methodology, Writing – review & editing. CG: Investigation, Writing – review & editing. YYu: Conceptualization, Investigation, Supervision, Writing – review & editing.

## Footnotes

^1^http://www.majorbio.com

## Supplementary material

Supplementary Figure S1 Effect of ageing on serum LBP level. Circulating serum LBP levels were measured by ELISA in both young and age mice. **p* < 0.05.

## References

[ref1] Acosta J. C. Banito A. Wuestefeld T. Georgilis A. Janich P. Morton J. P.. (2013). A complex secretory program orchestrated by the inflammasome controls paracrine senescence. Nat. Cell Biol. 15, 978–990. doi: 10.1038/ncb2784, PMID: 23770676 PMC3732483

[ref2] Biagi E. Franceschi C. Rampelli S. Severgnini M. Ostan R. Turroni S.. (2016). Gut microbiota and extreme longevity. Curr. Biol. 26, 1480–1485. doi: 10.1016/j.cub.2016.04.016, PMID: 27185560

[ref3] Bleve A. Motta F. Durante B. Pandolfo C. Selmi C. (2023). Immunosenescence, Inflammaging, and Frailty: Role of Myeloid Cells in Age-Related Diseases. Clin Rev Allergy Immunol 64, 123–144. doi: 10.1007/s12016-021-08909-7 35031957 PMC8760106

[ref4] Boehme M. Guzzetta K. E. Bastiaanssen T. F. S. van de Wouw M. Moloney G. M. Gual-Grau A.. (2021). Microbiota from young mice counteracts selective age-associated behavioral deficits. Nat Aging 1, 666–676. doi: 10.1038/s43587-021-00093-9, PMID: 37117767

[ref5] Borre Y. E. O'Keeffe G. W. Clarke G. Stanton C. Dinan T. G. Cryan J. F. (2014). Microbiota and neurodevelopmental windows: implications for brain disorders. Trends Mol. Med. 20, 509–518. doi: 10.1016/j.molmed.2014.05.002, PMID: 24956966

[ref6] Cai Y. Y. Huang F. Q. Lao X. Lu Y. Gao X. Alolga R. N.. (2022). Integrated metagenomics identifies a crucial role for trimethylamine-producing Lachnoclostridium in promoting atherosclerosis. NPJ Biofilms Microbiomes 8:11. doi: 10.1038/s41522-022-00273-4, PMID: 35273169 PMC8913745

[ref7] Campisi J. Kapahi P. Lithgow G. J. Melov S. Newman J. C. Verdin E. (2019). From discoveries in ageing research to therapeutics for healthy ageing. Nature 571, 183–192. doi: 10.1038/s41586-019-1365-2 31292558 PMC7205183

[ref8] Castellani G. Croese T. Peralta Ramos J. M. Schwartz M. (2023). Transforming the understanding of brain immunity. Science 380:eabo7649. doi: 10.1126/science.abo7649, PMID: 37023203

[ref9] Claesson M. J. Cusack S. O'Sullivan O. Greene-Diniz R. de Weerd H. Flannery E.. (2011). Composition, variability, and temporal stability of the intestinal microbiota of the elderly. Proc. Natl. Acad. Sci. U. S. A. 108, 4586–4591. doi: 10.1073/pnas.1000097107, PMID: 20571116 PMC3063589

[ref10] Conway J. Duggal N. A. (2021). Ageing of the gut microbiome: potential influences on immune senescence and inflammageing. Ageing Res. Rev. 68:101323. doi: 10.1016/j.arr.2021.101323, PMID: 33771720

[ref11] Cruz-Pereira J. S. Moloney G. M. Bastiaanssen T. F. S. Boscaini S. Fitzgerald P. Clarke G.. (2023). Age-associated deficits in social behaviour are microbiota-dependent. Brain Behav. Immun. 110, 119–124. doi: 10.1016/j.bbi.2023.02.008, PMID: 36791892

[ref12] Dixon W. J. (1980). Efficient analysis of experimental observations. Annu. Rev. Pharmacol. Toxicol. 20, 441–462. doi: 10.1146/annurev.pa.20.040180.002301 7387124

[ref13] Dong Y. Cui C. (2022). The role of short-chain fatty acids in central nervous system diseases. Mol. Cell. Biochem. 477, 2595–2607. doi: 10.1007/s11010-022-04471-8 35596843

[ref14] Du Y. Gao Y. Zeng B. Fan X. Yang D. Yang M. (2021). Effects of anti-aging interventions on intestinal microbiota. Gut Microbes 13:1994835. doi: 10.1080/19490976.2021.1994835, PMID: 34743658 PMC8583001

[ref15] Fane M. Weeraratna A. T. (2020). How the ageing microenvironment influences tumour progression. Nat. Rev. Cancer 20, 89–106. doi: 10.1038/s41568-019-0222-9 31836838 PMC7377404

[ref16] Fang E. F. Scheibye-Knudsen M. Jahn H. J. Li J. Ling L. Guo H.. (2015). A research agenda for aging in China in the 21st century. Ageing Res. Rev. 24, 197–205. doi: 10.1016/j.arr.2015.08.003, PMID: 26304837 PMC5179143

[ref17] Ghosh T. S. Shanahan F. O'Toole P. W. (2022). The gut microbiome as a modulator of healthy ageing. Nat. Rev. Gastroenterol. Hepatol. 19, 565–584. doi: 10.1038/s41575-022-00605-x 35468952 PMC9035980

[ref18] Grobler C. van Tongeren M. Gettemans J. Kell D. B. Pretorius E. (2023). Alzheimer's disease: a systems view provides a unifying explanation of its development. J. Alzheimers Dis. 91, 43–70. doi: 10.3233/JAD-220720 36442193

[ref19] Hsiao E. Y. McBride S. W. Hsien S. Sharon G. Hyde E. R. McCue T.. (2013). Microbiota modulate behavioral and physiological abnormalities associated with neurodevelopmental disorders. Cell 155, 1451–1463. doi: 10.1016/j.cell.2013.11.024, PMID: 24315484 PMC3897394

[ref20] Jing Y. Yang D. Bai F. Wang Q. Zhang C. Yan Y.. (2023). Yu, neurological recovery partly through short-chain fatty acids. NPJ Biofilms Microbiomes 9:99. doi: 10.1038/s41522-023-00466-5, PMID: 38092763 PMC10719379

[ref21] Kigerl K. A. Zane K. Adams K. Sullivan M. B. Popovich P. G. (2020). The spinal cord-gut-immune axis as a master regulator of health and neurological function after spinal cord injury. Exp. Neurol. 323:113085. doi: 10.1016/j.expneurol.2019.113085 31654639 PMC6918675

[ref22] Kim K. A. Jeong J. J. Yoo S. Y. Kim D. H. (2016). Gut microbiota lipopolysaccharide accelerates inflamm-aging in mice. BMC Microbiol. 16:9. doi: 10.1186/s12866-016-0625-7, PMID: 26772806 PMC4715324

[ref23] Leng F. Edison P. (2021). Neuroinflammation and microglial activation in Alzheimer disease: where do we go from here? Nat. Rev. Immunol. 17, 157–172. doi: 10.1038/s41582-020-00435-y 33318676

[ref24] Levy M. Kolodziejczyk A. A. Thaiss C. A. Elinav E. (2017). Dysbiosis and the immune system. Nat. Rev. Immunol. 17, 219–232. doi: 10.1038/nri.2017.7 28260787

[ref25] Li Y. Ning L. Yin Y. Wang R. Zhang Z. Hao L.. (2020). Age-related shifts in gut microbiota contribute to cognitive decline in aged rats. Aging 12, 7801–7817. doi: 10.18632/aging.103093, PMID: 32357144 PMC7244050

[ref26] Liu Y. Li H. Wang X. Huang J. Zhao D. Tan Y.. (2023). Anti-Alzheimers molecular mechanism of icariin: insights from gut microbiota, metabolomics, and network pharmacology. J. Transl. Med. 21:277. doi: 10.1186/s12967-023-04137-z, PMID: 37095548 PMC10124026

[ref27] Lv W. J. Liu C. Yu L. Z. Zhou J. H. Li Y. Xiong Y.. (2020). Melatonin alleviates neuroinflammation and metabolic disorder in DSS-induced depression rats. Oxid. Med. Cell. Longev. 2020:1241894. doi: 10.1155/2020/1241894 32802257 PMC7415091

[ref28] Mabbott N. A. (2015). A breakdown in communication? Understanding the effects of aging on the human small intestine epithelium. Clin. Sci. 129, 529–531. doi: 10.1042/CS20150364, PMID: 26186738 PMC4613503

[ref29] Man A. L. Gicheva N. Nicoletti C. (2014). The impact of ageing on the intestinal epithelial barrier and immune system. Cell. Immunol. 289, 112–118. doi: 10.1016/j.cellimm.2014.04.001, PMID: 24759078

[ref30] Marschallinger J. Iram T. Zardeneta M. Lee S. E. Lehallier B. Haney M. S.. (2020). Author correction: lipid-droplet-accumulating microglia represent a dysfunctional and proinflammatory state in the aging brain. Nat. Neurosci. 23:1308. doi: 10.1038/s41593-020-0682-y, PMID: 32719564

[ref31] Matcovitch-Natan O. Winter D. R. Giladi A. Vargas Aguilar S. Spinrad A. Sarrazin S.. (2016). Microglia development follows a stepwise program to regulate brain homeostasis. Science 353:aad8670. doi: 10.1126/science.aad8670 27338705

[ref32] Medina-Rodriguez E. M. Watson J. Reyes J. Trivedi M. Beurel E. (2023). Th17 cells sense microbiome to promote depressive-like behaviors. Microbiome 11:92. doi: 10.1186/s40168-022-01428-3, PMID: 37106375 PMC10142784

[ref33] Mossad O. Batut B. Yilmaz B. Dokalis N. Mezo C. Nent E.. (2022). Gut microbiota drives age-related oxidative stress and mitochondrial damage in microglia via the metabolite N(6)-carboxymethyllysine. Nat. Neurosci. 25, 295–305. doi: 10.1038/s41593-022-01027-3, PMID: 35241804

[ref34] O'Hara A. M. Shanahan F. (2006). The gut flora as a forgotten organ. EMBO Rep. 7, 688–693. doi: 10.1038/sj.embor.7400731, PMID: 16819463 PMC1500832

[ref35] Pajares M. Manda I. Boscá L. Cuadrado A. (2020). Inflammation in Parkinson's disease: mechanisms and therapeutic implications. Cells 9:1687. doi: 10.3390/cells9071687, PMID: 32674367 PMC7408280

[ref36] Pang Y. Zheng Y. Yang N. Zan M. Zhang L. Ding W. (2022). Potential novel biomarkers in small intestine for obesity/obesity resistance revealed by multi-omics analysis. Lipids Health Dis. 21:98. doi: 10.1186/s12944-022-01711-0, PMID: 36209126 PMC9547412

[ref37] Parker A. Romano S. Ansorge R. Aboelnour A. Le Gall G. Savva G. M.. (2022). Fecal microbiota transfer between young and aged mice reverses hallmarks of the aging gut, eye, and brain. Microbiome 10:68. doi: 10.1186/s40168-022-01243-w, PMID: 35501923 PMC9063061

[ref38] Russell A. Copio J. N. Shi Y. Kang S. Franklin C. L. Ericsson A. C. (2022). Reduced housing density improves statistical power of murine gut microbiota studies. Cell Rep. 39:110783. doi: 10.1016/j.celrep.2022.110783 35545042 PMC9161176

[ref39] Sadler R. Cramer J. V. Heindl S. Kostidis S. Betz D. Zuurbier K. R.. (2020). Short-chain fatty acids improve poststroke recovery via immunological mechanisms. J. Neurosci. 40, 1162–1173. doi: 10.1523/JNEUROSCI.1359-19.2019, PMID: 31889008 PMC6989004

[ref40] Salazar N. Arboleya S. Fernandez-Navarro T. de Los Reyes-Gavilan C. G. Gonzalez S. Gueimonde M. (2019). Age-associated changes in gut microbiota and dietary components related with the immune system in adulthood and old age: a cross-sectional study. Nutrients 11:1765. doi: 10.3390/nu11081765, PMID: 31370376 PMC6722604

[ref41] Sampson T. R. Debelius J. W. Thron T. Janssen S. Shastri G. G. Ilhan Z. E.. (2016). Gut microbiota regulate motor deficits and neuroinflammation in a model of Parkinson's disease. Cell 167, 1469–1480.e12. doi: 10.1016/j.cell.2016.11.018, PMID: 27912057 PMC5718049

[ref42] Santoro A. Ostan R. Candela M. Biagi E. Brigidi P. Capri M.. (2018). Gut microbiota changes in the extreme decades of human life: a focus on centenarians. Cell. Mol. Life Sci. 75, 129–148. doi: 10.1007/s00018-017-2674-y 29032502 PMC5752746

[ref43] Scott K. A. Ida M. Peterson V. L. Prenderville J. A. Moloney G. M. Izumo T.. (2017). Revisiting Metchnikoff: age-related alterations in microbiota-gut-brain axis in the mouse. Brain Behav. Immun. 65, 20–32. doi: 10.1016/j.bbi.2017.02.004, PMID: 28179108

[ref44] Shin J. Noh J. R. Choe D. Lee N. Song Y. Cho S.. (2021). Ageing and rejuvenation models reveal changes in key microbial communities associated with healthy ageing. Microbiome 9:240. doi: 10.1186/s40168-021-01189-5, PMID: 34906228 PMC8672520

[ref45] Sierra A. Encinas J. M. Deudero J. J. Chancey J. H. Enikolopov G. Overstreet-Wadiche L. S.. (2010). Microglia shape adult hippocampal neurogenesis through apoptosis-coupled phagocytosis. Cell Stem Cell 7, 483–495. doi: 10.1016/j.stem.2010.08.014, PMID: 20887954 PMC4008496

[ref46] Silva Y. P. Bernardi A. Frozza R. L. (2020). The role of short-chain fatty acids from gut microbiota in gut-brain communication. Front. Endocrinol. 11:25. doi: 10.3389/fendo.2020.00025, PMID: 32082260 PMC7005631

[ref47] Socodato R. Portugal C. C. Canedo T. Rodrigues A. Almeida T. O. Henriques J. F.. (2020). Microglia dysfunction caused by the loss of Rhoa disrupts neuronal physiology and leads to neurodegeneration. Cell Rep. 31:107796. doi: 10.1016/j.celrep.2020.107796 32579923

[ref48] Sun P. Wang M. Liu Y. X. Li L. Chai X. Zheng W.. (2023). High-fat diet-disturbed gut microbiota-colonocyte interactions contribute to dysregulating peripheral tryptophan-kynurenine metabolism. Microbiome 11:154. doi: 10.1186/s40168-023-01606-x, PMID: 37468922 PMC10355067

[ref49] Tan J. Dai A. Pan L. Zhang L. Wang Z. Ke T.. (2021). Inflamm-aging-related cytokines of IL-17 and IFN-γ accelerate Osteoclastogenesis and periodontal destruction. J. Immunol. Res. 2021:9919024. doi: 10.1155/2021/9919024 34395635 PMC8357511

[ref50] Tansey M. G. Wallings R. L. Houser M. C. (2022). Inflammation and immune dysfunction in Parkinson disease. Nat. Rev. Immunol. 22, 657–673. doi: 10.1038/s41577-022-00684-6 35246670 PMC8895080

[ref51] Tavella T. Rampelli S. Guidarelli G. Bazzocchi A. Gasperini C. Pujos-Guillot E.. (2021). Elevated gut microbiome abundance of Christensenellaceae, Porphyromonadaceae and Rikenellaceae is associated with reduced visceral adipose tissue and healthier metabolic profile in Italian elderly. Gut Microbes 13, 1–19. doi: 10.1080/19490976.2021.1880221, PMID: 33557667 PMC7889099

[ref52] Thevaranjan N. Puchta A. Schulz C. Naidoo A. Szamosi J. C. Verschoor C. P.. (2018). Age-associated microbial dysbiosis promotes intestinal permeability, systemic inflammation, and macrophage dysfunction. Cell Host Microbe 23:570. doi: 10.1016/j.chom.2018.03.006, PMID: 29649447 PMC5899819

[ref53] Thion M. S. Low D. Silvin A. Chen J. Grisel P. Schulte-Schrepping J.. (2018). Microbiome influences prenatal and adult microglia in a sex-specific manner. Cell 172, 500–516.e16. doi: 10.1016/j.cell.2017.11.042, PMID: 29275859 PMC5786503

[ref54] Tian B. Geng Y. Wang P. Cai M. Neng J. Hu J.. (2022). Ferulic acid improves intestinal barrier function through altering gut microbiota composition in high-fat diet-induced mice. Eur. J. Nutr. 61, 3767–3783. doi: 10.1007/s00394-022-02927-7, PMID: 35732902

[ref55] Wang J. Zhong Y. Zhu H. Mahgoub O. K. Jian Z. Gu L.. (2022). Different gender-derived gut microbiota influence stroke outcomes by mitigating inflammation. J. Neuroinflammation 19:245. doi: 10.1186/s12974-022-02606-8, PMID: 36195899 PMC9531521

[ref56] Wu M. D. Montgomery S. L. Rivera-Escalera F. Olschowka J. A. O'Banion M. K. (2013). Sustained IL-1beta expression impairs adult hippocampal neurogenesis independent of IL-1 signaling in nestin+ neural precursor cells. Brain Behav. Immun. 32, 9–18. doi: 10.1016/j.bbi.2013.03.003, PMID: 23510988 PMC3686979

[ref57] Xiao W. Su J. Gao X. Yang H. Weng R. Ni W.. (2022). The microbiota-gut-brain axis participates in chronic cerebral hypoperfusion by disrupting the metabolism of short-chain fatty acids. Microbiome 10:62. doi: 10.1186/s40168-022-01255-6 35430804 PMC9013454

[ref58] Yang L. Wang Y. Li Z. Wu X. Mei J. Zheng G. (2023). Brain targeted peptide-functionalized chitosan nanoparticles for resveratrol delivery: impact on insulin resistance and gut microbiota in obesity-related Alzheimer's disease. Carbohydr. Polym. 310:120714. doi: 10.1016/j.carbpol.2023.120714, PMID: 36925241

[ref59] Ye X. Liu Y. Hu J. Gao Y. Ma Y. Wen D. (2021). Chlorogenic acid-induced gut microbiota improves metabolic endotoxemia. Front. Endocrinol. 12:762691. doi: 10.3389/fendo.2021.762691, PMID: 34975748 PMC8716487

[ref60] Zafar H. Saier M. H. Jr. (2021). Gut Bacteroides species in health and disease. Gut Microbes 13, 1–20. doi: 10.1080/19490976.2020.1848158, PMID: 33535896 PMC7872030

[ref61] Zareie P. Connor B. La Flamme A. C. (2017). Amelioration of experimental autoimmune encephalomyelitis by clozapine is not associated with defective CD4 T cell responses. J. Neuroinflammation 14:68. doi: 10.1186/s12974-017-0842-5, PMID: 28356108 PMC5372297

[ref62] Zeng X. Li J. Shan W. Lai Z. Zuo Z. (2023). Gut microbiota of old mice worsens neurological outcome after brain ischemia via increased valeric acid and IL-17 in the blood. Microbiome 11:204. doi: 10.1186/s40168-023-01648-1, PMID: 37697393 PMC10496352

[ref63] Zhu X. Sakamoto S. Ishii C. Smith M. D. Ito K. Obayashi M.. (2023). Dectin-1 signaling on colonic gammadelta T cells promotes psychosocial stress responses. Nat. Immunol. 24, 625–636. doi: 10.1038/s41590-023-01447-8, PMID: 36941398 PMC10704545

