# Supplementary figures and images for: Role of microbiota-gut-brain axis in natural aging-related alterations in behavior
Source: Front Neurosci. 2024 Apr 18;18:1362239. doi: 10.3389/fnins.2024.1362239 (PMC11063250; doi:10.3389/fnins.2024.1362239)

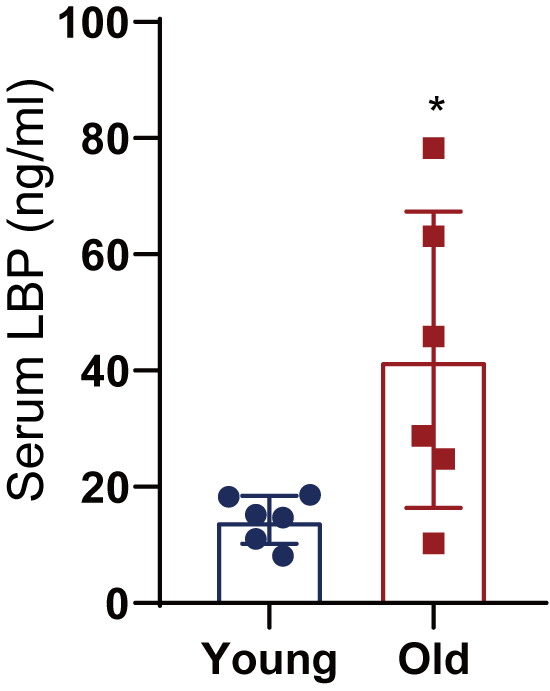

Supplement: Supplementary Figure S1 — Effect of ageing on serum LBP level. Circulating serum LBP levels were measured by ELISA in both young and age mice. *p < 0.05. [file Image_1.tif]
